# Meteorological factors and risk of scrub typhus in Guangzhou, southern China, 2006–2012

**DOI:** 10.1186/1471-2334-14-139

**Published:** 2014-03-12

**Authors:** Tiegang Li, Zhicong Yang, Zhiqiang Dong, Ming Wang

**Affiliations:** 1 Guangzhou Center for Disease Control and Prevention, No 1, Qide Rd, Jiahe, Baiyun District, Guangzhou, Guangdong Province 510440, China

**Keywords:** Scrub typhus, Meteorological variables, Risk factor, Correlation, Early warning

## Abstract

**Background:**

Scrub typhus is becoming the most common vector born disease in Guangzhou, southern China. In this study, we aimed to examine the effect of weather patterns on the incidence of Scrub typhus in the subtropical city of Guangzhou for the period 2006–2012, and assist public health prevention and control measures.

**Methods:**

Scrub typhus reported cases during the period of 2006–2012 in Guangzhou were obtained from National Notifiable Disease Report System (NNDRS). Simultaneous meteorological data including temperature, relative humidity, atmospheric pressure, sunshine, and rainfall were obtained from the documentation of the Guangzhou Meteorological Bureau. A negative binomial regression was used to identify the relationship between meteorological variables and scrub typhus.

**Results:**

Annual incidence rates of scrub typhus from 2006 to 2012 were 3.25, 2.67, 3.81, 4.22, 4.41, 5.12, and 9.75 (per 100 000) respectively. Each 1°C rise in temperature corresponded to an increase of 14.98% (95% CI 13.65% to 16.33%) in the monthly number of scrub typhus cases, while a 1 hPa rise in atmospheric pressure corresponded to a decrease in the number of cases by 8.03% (95% CI −8.75% to −7.31%). Similarly, a 1 hour rise in sunshine corresponded to an increase of 0.17% or 0.54%, and a 1 millimeter rise in rainfall corresponded to an increase of 0.05% or 0.10%, in the monthly number of scrub typhus cases, depending on the variables considered in the model.

**Conclusion:**

Our study provided evidence that climatic factors were associated with occurrence of scrub typhus in Guangzhou city, China. Temperature, duration of sunshine, and rainfall were positively associated with scrub typhus incidence, while atmospheric pressure was inversely associated with scrub typhus incidence. These findings should be considered in the prediction of future patterns of scrub typhus transmission.

## Background

Scrub typhus is a rickettsial disease caused by *Orientia tsutsugamushi*[1], which is transmitted to humans through infected chigger mites. When the rickettsia is transmitted through the bite of an infected mite to human, it begins to proliferate at the bite site and a characteristic skin lesion, known as an eschar, is formed. The pathogen then spreads systemically via the hematogenous and lymphogenous routes. Infected people develop various systemic symptoms and reactions including fever, cutaneous rash, lymphadenopathy, elevations of C-reacting protein (CRP) and liver enzymes [2,3].

Globally, scrub typhus is widely distributed in southern Asia [4], in a triangle from northern Japan and far-eastern Russia in the north to northern Australia in the south and to Pakistan and Afghanistan in the west, as well as in the islands of the western Pacific and Indian Oceans. More than half (55%) of the world’s population lives in areas where scrub typhus is endemic, approximately 1 billion persons are estimated at risk for the disease [5]. In China, the first scrub typhus case was reported in 1948 in Guangzhou [6]. To control and prevention of scrub typhus, Guangzhou government has legislated the inclusion of scrub typhus into local reportable disease inventory since 1995. This means, as for other national reportable disease, physicians who diagnose suspected or confirmed scrub typhus cases must report these cases to Guangzhou Centers for Disease Control and Prevention (GZCDC) via the National Notifiable Disease Report System (NNDRS). The reported cases to the local health department showed that the incidence of scrub typhus has a rapid increasing trend in Guangzhou. In 2012, a total of 1252 scrub typhus were reported, which accounted for the greatest proportion of vector borne diseases reported, leading the public health authorities to concern the increased incidence

Currently, effective chemoprophylaxis or vaccination approaches for dealing with scrub typhus are still not available [7]. Programs to prevent this disease concentrate on monitoring and predicting scrub typhus incidence. In recent decades, weather variables including temperature and humidity have been widely studied for their potential as early warning tools to fend off climate-sensitive infectious diseases such as fecal-oral infection disease [8], malaria [9], respiratory tract infectious [10], and Dengue fever [11]. Previous studies have reported that the incidence of scrub typhus exhibited seasonal variation in a number of different areas. For example, in Korea, scrub typhus outbreak increased drastically in October, peaked in November and started to decrease in December when the coldest season begins. In Japan, the scrub typhus infections are typically present throughout the year, showing small peaks in autumn and winter [12]. Moreover, even in China, the scrub typhus infection showed different epidemic types in different areas. In Northern province of Shandong, monthly changes in the number of scrub typhus cases indicated an epidemic period from September to November, with a peak in October [13], While in Southern province of Guangdong, a bimodal seasonal pattern has been detected, which is characterized by a large peak in June and a small peak in September [14]. The seasonality of scrub typhus suggests that meteorological variables might be influential in the spread of the disease.

Very little information is available for understanding the relationship between meteorological variables and scrub typhus, and the published literatures indicated inconsistent findings. For example, a recent study in Taiwan [15] found a non-significant association between rainfall and scrub typhus, in contrast to findings in Thailand [16]. In addition, longer hours of sunshine was shown to be a risk factor for scrub typhus in the Taiwan study [17], but no other study supported these findings. Therefore, there is an urgent need to investigate the relationship between meteorological variables and scrub typhus which can help in establishing the development of an early warning system for scrub typhus.

In this study, we aimed to estimate the effects of diverse climate variables, such as temperature, relative humidity, rainfall, and air pressure on the incidence of scrub typhus in the subtropical city of Guangzhou from 2006–2012, in order to assist the prevention and control of scrub typhus.

## Methods

### Study area

Guangzhou is 7434.4 square kilometers in size, situated in north latitude 22°26'N to 23°56'N and east longitude 112°57'E to 114°3'E, with over 7.94 million registered inhabitants and 4.76 million floating population (from 2010 census data). It traverses the Tropic of Cancer, and the climate is characterized as humid subtropical and influenced by the Asian monsoon. Summers are wet with high temperatures and a high humidity index. Winters are mild, dry and sunny. The annual mean temperature ranges from 18°C to 25°C. The annual rainfall is typically between 1500 mm and 2000 mm.

### Surveillance data of scrub typhus

In this study, we obtained data of scrub typhus cases in Guangzhou from NNDRS during the period of 2006–2012. In China, all cases of scrub typhus were diagnosed according to the unified diagnostic criteria issued by Chinese Ministry of Health (MOH). The criteria for a probable case of scrub typhus include epidemiological exposure histories (traveling to an endemic area and contact with chiggers or rodents within three weeks before the onset of illness), clinical manifestations (such as high fever, lymphadenopathy, skin rash, and eschars or ulcers), and an agglutination titer ≥1:160 in the Weil-Felix test using the OXK strain of *Proteus mirabilis*. The case definition of confirmed scrub typhus must fulfill the above criteria for a probable case and also meet at least one of the laboratory criteria for confirmatory diagnosis: a fourfold or greater rise in serum IgG antibody titers between acute and convalescent sera detected by using indirect immunofluorescence antibody assay (IFA), detection of *O. tsutsugamushi* by polymerase chain reaction (PCR) in clinical specimens, or isolation of *O. tsutsugamushi* from clinical specimens [18]. All the laboratory tests were completed by Guangzhou CDC using the same method and same kits. A standard form was adopted by local physicians and epidemiologist to collect individual information on each scrub typhus case, including age, address, date of onset, diagnosis, and laboratory test results. Routine case reporting is done by hospitals via NNDRS within 24 hours. The cases from whole city of Guangzhou and confirmed by laboratory were used in this study.

### Meteorological data

Simultaneous meteorological data, including daily average temperature (in degrees Centigrade), relative humidity (as a percentage), atmospheric pressure (in hPa), wind velocity (in meters per second), sunshine (in hours of daylight) and rainfall (in millimeter) were obtained from the documentation of the Guangzhou Meteorological Bureau. The weather data were measured at a fixed-site station located in a central district of Guangzhou (in latitude of 23.120608 and longitude of 113.26812). Meteorological instruments included barometers, pressure readings, thermometers, anemometers, actinometers, psychrometers, evaporimeters, and weather vanes. The measurements of temperature, relative humidity, atmospheric pressure and wind velocity were usually taken every 3 h before the daily average was calculated. However, for sunshine and rainfall, the daily total was used.

### Data analysis

A negative binomial multivariable regression was used to explore the relationship between meteorological variables and scrub typhus. Negative binomial distribution is a Poisson distribution with an extra-dispersion term, the extra dispersion term acts as a random effect that subjects the Poisson means to additional variation that has a gamma distribution. Given the data were over-dispersed; we chose a negative binomial distribution model rather than a Poisson model. The cases were typhus occurrence. Data was presented as the incidence of scrub typhus per 100 000 inhabitants grouped by month of onset. The meteorological variables were calculated by monthly average or aggregate. A preliminary analysis was conducted through Pearson’s correlation coefficient (r) matrix within meteorological variables. This indicated that the model constructed using contemporaneously both temperature and atmospheric pressure suffered from collinearity problems, because the two variables showed strong negative correlation (r = −0.86, P < 0.01). Thus, we conducted two separate negative binomial regression models: the first included average temperature but no atmospheric pressure, while the second included atmospheric pressure but no temperature. Both models included additionally relative humidity, wind velocity, sunshine and rainfall as independent variables. To quantify the effects of meteorological variables, we computed the influences (e^β^ − 1)* 100, which virtually correspond to the percent increase. The final model included only those variables that reached a *P* value of <0 · 05. However, in order to control the yearly fluctuant, the ‘year’ variable was forced into the final model even though it was not significant. These analyses were performed using SAS (V.8.01, SAS Institute, Cary, New Jersey, USA). *P* values <0.05 were considered statistically significant.

### Ethics statement

This study was approved by the ethics committee of Guangzhou center for control and prevention (GZCDC).

## Results

**Figure 1:**
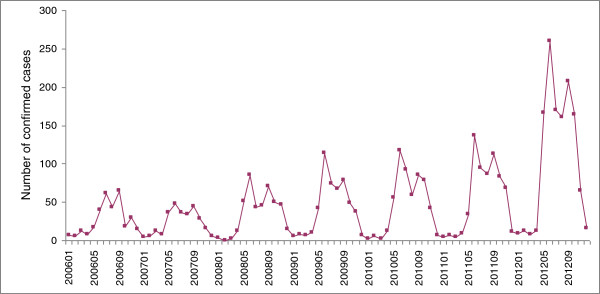
Scrub typhus confirmed cases in Guangzhou, southern China, 2006-2012.

From January 1, 2006 to December 31, 2012, a total of 3998 scrub typhus confirmed cases were reported in Guangzhou. Annual incidence rates from 2006 to 2012 were 3.25, 2.67, 3.81, 4.22, 4.41, 5.12, and 9.75 (per 100 000) respectively (Figure 1). Of particular note, in 2012 a total of 1252 confirmed cases were reported, which is 3.8 times the number reported in 2007 (324 cases). Monthly changes in the number of cases showed the epidemic peak occurred in May-October, 79.26% of total cases were reported during this period.

**Figure 2:**
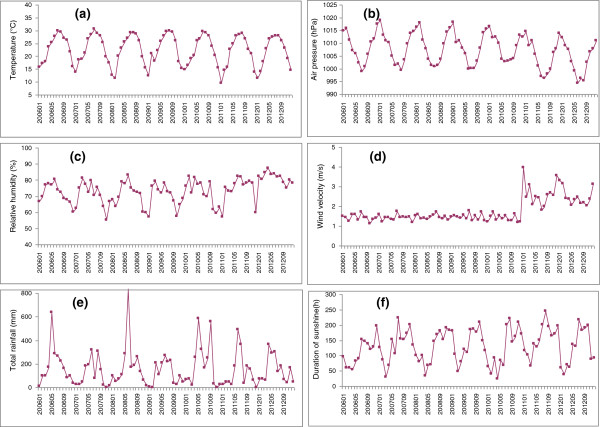
Monthly disturbance of a) average temperature; b) average atmospheric pressure; c) average relative humidity; d) average wind velocity; e)aggregate rainfall; f) aggregate sunshine in Guangzhou, southern China, 2006-2012.

During the study period, monthly minimum and maximum temperature was 9.52°C and 30.77°C, respectively, and average temperature was 22.49°C. The monthly relative humidity ranged from 55.10% to 87.33%, with an average of 73.20%. The monthly atmospheric pressure ranged from 994.28 hPa to 1018.90 hPa, with an average of 1007.29 hPa. The monthly wind velocity ranged from 1.12 m/s to 3.96 m/s, with an average of 1.76 m/s. The monthly aggregate rainfall ranged from 0.10 mm to 834.72 mm, with a median of 101.60 mm. The monthly aggregate sunshine ranged from 24.40 h to 245.00 h, with a median of 132.85 h. (Figure 2, Table 1).

**Table 1:** Summary statistics for monthly scrub typhus confirmed cases and weather conditions in Guangzhou, southern China, 2006-2012

	**Mean**	**S.D.**	**Min**	**P (25)**	**Median**	**P (75)**	**Max**
Average temperature (°C)	22.49	5.72	9.52	17.89	23.19	27.90	30.77
Average atmospheric pressure (hPa)	1007.29	6.18	994.28	1002.40	1007.83	1012.12	1018.90
Average relative humidity (%)	73.20	7.66	55.10	68.21	74.53	78.72	87.33
Average wind velocity (m/s)	1.76	0.60	1.12	1.37	1.51	2.03	3.96
Aggregate rainfall (mm)	156.10	156.64	0.10	43.77	101.60	222.62	834.72
Aggregate sunshine (h)	130.15	55.21	24.40	82.85	132.85	179.05	245.00
Scrub typhus confirmed cases	47.60	51.83	0.00	8.00	35.00	67.50	260.00

*Note. *all the data were presented as monthly average or aggregate values.

S.D. = Standard deviation.

The correlations between independent variables revealed a strong correlation (r = −0.86, P < 0.01) between average temperature and atmospheric pressure (Table 2). Therefore, to avoid collinearity problems, we decided to explore the relationship of temperature and atmospheric pressure in scrub typhus cases by using two different models, including either temperature (Table 3-Model A) or atmospheric pressure (Table 3-Model B) together with all other predictors. In the two models, temperature (p < 0.01) and atmospheric pressure (*p* < 0.01) were highly significant; sunshine, rainfall and year were also significant in both models (all *p* < 0.01). Table 3-Modle C and D included only those variables respectively from model A and B that reached a *P* value of <0 · 05. After adjusting by “year”, each 1°C rise in temperature corresponded to an increase of 14.98% (95% CI 13.65% to 16.33%) in the monthly number of scrub typhus cases, while a 1 hPa rise in atmospheric pressure corresponded to a decrease in the number of cases by 8.03% (95% CI −8.75% to −7.31%). Similarly, a 1 hour rise in sunshine corresponded to an increase of 0.17% or 0.54%, and a 1 millimeter rise in rainfall corresponded to an increase of 0.05% or 0.10%, in the monthly number of scrub typhus cases, depending on the variables considered in the model (Table 3-Model C and D). Rsqaure for final model C and D were 0.67 and 0.61, respectively.

**Table 2:** Pearson’s correlation coefficient (‘r’) matrix of meteorological variables in Guangzhou, southern China, 2006-2012

	**Atmospheric pressure**	**Relative humidity**	**Average temperature**	**Rainfall**	**Sunshine**	**Wind velocity**
Atmospheric pressure	1					
Relative humidity	−0.59 (p = 0.00)	1.00				
Average temp.	−0.86 (p = 0.00)	0.32 (p = 0.00)	1.00			
Rainfall	−0.58 (p = 0.00)	0.52 (p = 0.00)	0.53 (p = 0.00)	1.00		
Sunshine	-.028 (p = 0.01)	−0.43 (p = 0.00)	0.40 (p = 0.00)	−0.16 (p = 0.15)	1.00	
Wind velocity	−0.10 (p = 0.36)	0.20 (p = 0.06)	−0.28 (p = 0.01)	−0.12 (p = 0.29)	0.00 (p = 0.99)	1.00

**Table 3:** Negative binomial regression model of meteorological factors associated with risk of scrub typhus incidence in Guangzhou, southern China, 2006–2012*

	***β***	**S.E.**	***P***	**Percent increase = (e**^**β**^ − 1)*100	**95% CI for percent increase (%)**	
					**Lower boundary**	**Upper boundary**
**(A)**						
Intercept	−530.11	28.42	0.00	–	–	–
Aggregate sunshine	0.00	0.00	0.01	0.14	0.03	0.25
Aggregate rainfall	0.00	0.00	0.00	0.05	0.03	0.08
Average wind velocity	0.09	0.05	0.08	9.15	−0.92	20.25
Average relative humidity	0.00	0.00	0.33	−0.45	−1.33	0.45
Average temperature	0.15	0.01	0.00	15.61	14.00	17.24
Year	0.26	0.01	0.00	30.20	26.62	33.88
**(B)**						
Intercept	−307.62	26.69	0.00	–	–	–
Aggregate sunshine	0.00	0.00	0.00	0.40	0.29	0.50
Aggregate rainfall	0.00	0.00	0.00	0.08	0.06	0.11
Average wind velocity	−0.19	0.05	0.06	−17.20	−24.16	122.77
Average relative humidity	−0.01	0.03	0.17	−1.32	−2.33	102.00
Average atmospheric pressure	−0.10	0.01	0.00	−9.36	−10.38	−8.34
Year	0.20	0.01	0.00	22.70	19.47	26.02
**(C)**						
Intercept	−540.03	18.48	0.00	–	–	–
Aggregate sunshine	0.00	0.00	0.00	0.17	0.08	0.26
Aggregate rainfall	0.00	0.00	0.00	0.05	0.02	0.08
Average temperature	0.14	0.01	0.00	14.98	13.65	16.33
Year	0.27	0.01	0.00	30.84	28.50	33.22
**(D)**						
Intercept	−184.32	20.37	0.00	–	–	–
Aggregate sunshine	0.01	0.00	0.00	0.54	0.47	0.62
Aggregate rainfall	0.00	0.00	0.00	0.10	0.07	0.12
Average atmospheric pressure	−0.08	0.00	0.00	−8.03	−8.75	−7.31
Year	0.14	0.01	0.00	14.46	12.42	16.54

***Note.*** *Negative binomial regression model for monthly scrub typhus incidence without atmospheric pressure (A) and without average temperature (B). Final models without atmospheric pressure (C) and without average temperature (D).

CI = Confidence interval, S.E. = Standard error.

## Discussion

Weather factors such as temperature and humidity have been proved to have significant association with occurrence and transmission of some infectious diseases. For example, in Switzerland, higher water vapor pressure and heat were found to be associated with a higher risk of community-acquired Legionnaires’ disease (LD) [19]; in Botswana, the elevation of annual minimum temperature was considered as the critical factor for continuous ascent in the number of diarrheal disease reported during the period of 1974-2003 [20]. The result of current study showed that temperature was positively associated with scrub typhus incidence in Guangzhou, each 1°C rise in temperature corresponded to an increase of 14.98% in the monthly number of scrub typhus cases. This finding is in general agreement with other studies [21-23] in which temperature is considered to be a precipitating factor for scrub typhus transmission. It is reported that in Korea, majority of the scrub typhus cases occurred in the cool weather [12]. Lee IY and his work team found that the high incidence of scrub typhus in humans during October through December in Korea is due to high populations of *L. pallidum* and *L. scutellare* that peak from September through November, and which sharply decline and remain low throughout December through August [24]. In Guangzhou, *Leptotrombidium deliense* is the primarily specie of chigger and the main vectors of scrub typhus. The *Leptotrombidium deliense* usually causes summer epidemic type of scrub typhus. As we have previously reported that majority of scrub typhus cases occurred between June and September in Guangzhou [25]. These added the evidence that the temperature maybe a useful predictor of scrub typhus and the temperature influences the occurrence of human infection with scrub typhus probably by influencing the activities of chiggers. However, our finding may only apply to the area where the weather pattern is similar to Guangzhou and the temperature is not too hot. In hotter climates, the conclusion may be opposite. For example, Mathai E et al. found that the scrub typhus cases increased when the temperature cools down in southern India [26].

We found rainfall was positively associated with the scrub typhus incidence of the same month. Similar observation was also reported in neighbouring country of India [27] where a large number of cases were encountered during the period July to October, which receives the highest rainfall of 429–666 mm. in Thailand, Frances SP et al. found that the occurrence of *Leptotrombidium deliense* was influenced by rainfall, with more chiggers attached to rodents in the wetter months of the year [16]. Their study also showed the risk of exposure to infection with *O. tsutsugamushi* is greater during the wetter months of the year. Ambient humidity is the key factor that determines the prevalence and geographical distribution of chiggers. This is because water vapor in humid air is the main source of water for their survival. Clopton RE, et al. found that larval population densities of chiggers were greatest in areas of high humidity [28]. A reported from Chile reveled that chiggers survive and thrive well at relative humidity above 50% but desiccate and die at relative humilities below this [29]. A recent study in Korea showed that the incidence of scrub typhus was positively correlated with humidity [30]. Although we also examined the effects of relative humidity on scrub typhus incidence, no evidence of an association with the number of scrub typhus cases was detected. This discrepancy might be due to the effects of rainfall, which were controlled for in the present study, but not in the study conducted in Korea. These suggested that the association between rainfall and scrub typhus indicated by our study might base on the relative humidity ranged from 55.10% to 87.33%.

Similar to Tsai PJ [17] et al. we found the duration of sunshine was positively associated with incidence of scrub typhus, and each one hour rise in sunshine led to an increase of 0.17% or 0.54% in the monthly number of scrub typhus cases. Previous study revealed that chigger activity correlated with a microclimatically driven diurnal rhythm [16], activity was greatest during afternoon-sundown, and dropped to low levels in the evening and remained so until sunrise. Furthermore, longer duration of sunshine probably corresponds to more time for human outdoors activities and more frequent visitation to forest lands or grass lands [17]. As a result, more likely to expose to infected mites, leading to an increase of scrub typhus infection [15,31].

To the best of our knowledge, the relationship between atmospheric pressure and scrub typhus has not been reported. The present report is first to investigate the effect of atmospheric pressure on scrub typhus incidence in southern China. We found that atmospheric pressure was inversely associated with the scrub typhus incidence of the same month. A possible explanation for this might be that high atmospheric pressure is adverse to mite survival [32]. In addition, as indicated by our study, the atmospheric pressure has a strong negative correlation with temperature. Although there was independent association between numbers of scrub typhus cases and increasing temperature and also reducing atmospheric pressure, these may represent the changes in the same season and are themselves associated. Therefore, continuing studies, which take these variables into account, will need to be undertaken.

Some limitations must be acknowledged for this study: Firstly, the incubation period of 10–12 days for every case cannot be determined exactly. We thus chose to use monthly aggregated data of scrub typhus and monthly average or aggregate meteorological data, the direction of these approximations are likely to be random, suggesting that our risk estimates are reliable. Secondly, our study did not further conduct the cross-correlation analysis of weather factors and scrub typhus, we could not exactly measure the lag influence of every weather factor. Thirdly, we obtained the weather data from only one weather station of Guangzhou, some description of the area under study such as rural, town, vegetation etc., can not be conducted in current study. Fourthly, owing to this investigation being an ecological study, although we emphasized the impact of climate, we could not exclude potential confounding factors. For example, our data demonstrated that the incidence rate of scrub typhus ranged from 2.67 to 5.12 per 100,000 populations from 2006 to 2011, but suddenly increased to 9.75 per 100,000 populations in 2012. Although our explanation is that these changes are correlated to the rise of temperature and atmosphere pressure, changes in temperature and atmosphere pressure in 2012 were not significant comparing to previous years. A possible explanation for this might be due to the fact that several Dengue fever (DF) outbreaks occurred in Guangzhou [11], a total of 172 DF confirmed cases were reported in 2012, which is 4.9 times the number reported in 2007. Public health authorities are concerned about its increased incidence and many work related to health education and health promotion were conducted and enhanced by health department, which may eventually increase the public awareness of vector disease in Guangzhou. In addition, human activity may be another important factor that influences the incidence of scrub typhus, as well as socio-economic factors, personal hygiene awareness etc. These need to be addressed in further studies.

Global climate change has profound impacts on infectious disease. Elucidation of the effects of weather variability on the epidemiology of infectious diseases is becoming important for disease control by public health officials and practitioners. The results of this study may aid in the prediction of epidemics and in preparation for the effects of climate changes on the epidemiology of scrub typhus through implementation of preventive public health interventions, such as promoting good hygiene practices, campaigns that include press releases and media events to encourage preventive activities (e.g. reducing the contacting with animal pets, having a good self-protection when going to vegetated areas). It is expected that such activities might be practically useful for preventing or limiting the spread of scrub typhus infections. Of course, some findings in the present study require confirmation, especially in different areas with different weather patterns.

## Conclusions

In conclusion, we reported that weather factors had significant association with occurrence and transmission of scrub typhus in Guangzhou, Southern China. A rise in temperature, duration of sunshine, and rainfall may increase the risk of scrub typhus infection, while an increase in atmospheric pressure may reduce the risk of scrub typhus infection. Our finding provided preliminary but fundamental information that may be useful for developing an early warning system.

## Competing interests

The authors declare no financial, academic or intellectual competing interests.

## Authors’ contributions

Conceived and designed the study: ZY, MW. Analyzed the data: TL, ZD. Contributed materials/analysis tools: ZY, MW. Wrote the paper: TL, ZY, MW. All authors contributed to and approved the final version of the manuscript.

## Pre-publication history

The pre-publication history for this paper can be accessed here:

http://www.biomedcentral.com/1471-2334/14/139/prepub
